# Single‐worm quantitative proteomics reveals aging heterogeneity in isogenic *Caenorhabditis elegans*

**DOI:** 10.1111/acel.14055

**Published:** 2023-12-03

**Authors:** Tian‐Yi Zhu, Shang‐Tong Li, Dan‐Dan Liu, Xiajun Zhang, Lianqi Zhou, Rong Zhou, Bing Yang

**Affiliations:** ^1^ Zhejiang Provincial Key Laboratory for Cancer Molecular Cell Biology, Life Sciences Institute Zhejiang University Hangzhou China; ^2^ Cancer Center Zhejiang University Hangzhou China; ^3^ Glbizzia Biosciences Co., Ltd Beijing China; ^4^ Institute of Animal Sciences Chinese Academy of Agricultural Sciences Beijing China

**Keywords:** aging, mass spectrometry, single worm proteomics

## Abstract

The heterogeneity of aging has been investigated at cellular and organic levels in the mouse model and human, but the exploration of aging heterogeneity at whole‐organism level is lacking. *C. elegans* is an ideal model organism for studying this question as they are self‐fertilized and cultured in the same chamber. Despite the tremendous progress made in single‐cell proteomic analysis, there is few single‐worm proteomics studies about aging. Here, we apply single‐worm quantitative mass spectrometry to quantify the heterogenous proteomic changes during aging across individuals, a total of 3524 proteins from 157 *C. eleagns* individuals were quantified. A reconstructed *C. elegans* aging trajectory and proteomic landscape of fast‐aging individuals were used to analyze the heterogeneity of *C. elegans* aging. We characterized inter‐individual proteomic variation during aging and revealed contributing factors that distinguish fast‐aging individuals from their siblings.

## Graphical abstract

This study quantitatively analyzed the proteomes of more than 150 worms during the aging process with a high temporal resolution. A reconstructed *C*. *elegans* aging trajectory reveals the dynamic aging process with heterogeneity among individuals. We characterized inter‐individual proteomic variation among isogenic individuals and revealed contributing factors that distinguish fast‐aging individuals from their siblings.

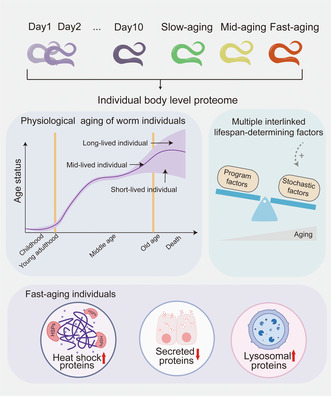

**Abbreviations**
ANOVA: analysis of variance
BP: biological process
CC: cellular component
CV: coefficient of variances
DEPs: differentially expressed proteins
GO: gene ontology
KEGG: kyoto encyclopedia of genes and genomes
LFQ: label free quantitation
MF: molecular function
MS: mass spectrometry
PCA: principal component analysis
ROS: reactive oxygen species

## 1 INTRODUCTION

Lifespan varies greatly among different species (Jones et al., 2014), which is mainly caused by genetic factors. The plasticity of lifespan not only appears in different species, but also exist among individuals in the same species under similar living conditions (Ruby et al., 2018; Stroustrup et al., 2016; Zhang et al., 2016). Despite the tremendous progress made in the genetic study of aging (Kenyon, 2010), little is known about how differences in lifespan arise within the same population. The lifespan of an individual could be affected by genetic, environmental and stochastic factors, accumulating evidence has shown the stochastic factors contributed substantially to lifespan (Gladyshev, 2016; Herndon et al., 2002; Ljungquist et al., 1998). However, the detailed aging events and lifespan variation caused by stochastic factors have not been dissected.

The self‐fertile hermaphrodite *C. elegans* is an ideal model to investigate the stochastic component in the aging process. The genomes are identical among a laboratory cultured *C. elegans* population, and the lifespan of genetically identical wild‐type (N2 Bristol) *C. elegans* varies from 1 week–4 weeks in the same cultural environment. The aging heterogeneity has been studied in several tissues and cell types in human and mouse models (Castillo‐Fernandez et al., 2020; Xia et al., 2020), however, the whole‐body molecular features are lacking. Besides, although it is difficult to track the aging process and future lifespan of worm individuals, worms with a slow movement rate in elder age have a significantly shorter lifespan, enabling us to pick up fast‐ and slow‐aging individuals for further proteomic analysis (Herndon et al., 2002).

Aging was long assumed to be a passive and stochastic process caused by random damage accumulation, however, such theories fail to explain the program‐like features of the aging process. The programmed theory of aging considered the aging process as a genetic program that evolved to cause aging (Bahar et al., 2006), but no such genes or genetic program have been identified whose specific role was to cause aging. It is still largely under debate about whether the aging process is programmed or stochastic. Measuring the aging process in *C. elegans* at individual level provides an opportunity to analyze both the programmed (age‐related changes at the population level) and stochastic (inter‐individual differences in isogenic population) components during the aging process.

Term of ‘single‐cell proteomics’ was first published on 2004 (Hemstreet & Wang, 2004), and then fluorescence based flow cytometry, mass cytometry and liquid chromatography tandem mass spectrometry (LC–MS/MS) were applied in single‐cell proteomics studies (Tajik et al., 2022). With the innovations in samples preparation and mass spectrometry instrumentation, more than 1000 protein groups can be identified from single HeLa cells (Cong et al., 2020), Advancements of single‐cell proteomics enable analyzing *C. elegans* proteome at single worm level (Bensaddek et al., 2016; Steinbach et al., 2022), but there is few aging related single worm proteomics study.

Here, we performed quantitative proteomic analyses on more than 150 worm individuals of different ages and aged individuals with different mobility. The analysis of inter‐individual variations helps dissect the programmed and stochastic components during the aging process, and key proteins underlying the lifespan plasticity among the genetically identical worm individuals.

## 2 METHODS

### 2.1 Age synchronization and determination of class A, B and C

To age‐synchronize worms, more than 1000 gravid adults were plated on five plates to lay eggs for 2 h and then removed; The young adult worms were considered to be day 1 and were transferred to new plates with 10 mM fluorodeoxyuridine (FUdR) at this time. Single worm from different ages were picked daily. Class A, B, and C were scored and picked on day 10 as described previously (Herndon et al., 2002). In brief, animals that moved well were scored as Class A, animals showing erratic movements were scored as Class B, and animals that showed little or no movement were scored as Class C. Scores for dead animals were based on their inability to respond to poking with a wire and their lack of pharyngeal pumping.

### 2.2 Protein extraction and proteolytic digestion in solution

For protein extraction, a single worm was picked and placed immediately into microfuge tubes containing 5 μL of lysis buffer (8 M urea in 100 mM triethyl ammonium bicarbonate (TEAB) pH 8.5, 15 mM tris‐carboxyethylphosphine (TCEP, Sigma) and flash frozen using liquid nitrogen. They were then thawed, centrifuged briefly, ultrasonicated using a sonicator (Qsonica, 80% power, 30 cycles: 30 s on, 30 s off), then alkylated in the dark for 15 min with 30 mM iodoacetamide (Sigma). The lysates were diluted with 100 mM TEAB to 2 M urea for digestion with trypsin. Trypsin (Promega) was used at an enzyme to substrate ratio of 1:25 (w/w). The digestions were carried out overnight at 37°C, then stopped by acidification with formic acid (FA) to a final concentration of 5% (v:v). Peptides were desalted using Empore C18 (3 M) following the manufacturer's instructions (Rappsilber et al., 2007), dried and redissolved in 0.1% FA for further LC–MS/MS analysis.

### 2.3 Mass spectrometry method

Peptides were separated on C18 (75 × 15 cm, 1.9 μm C18, 1 μm tip) with Easy‐nLC 1200 system. Using the following mobile phases: 2% ACN incorporating 0.1% FA (Solvent A) and 80% ACN incorporating 0.1% FA (Solvent B). Samples were analyzed with a 60 min gradient at a flow rate of 300 nL/min as follows: 4%–5% B for 1 min, 5%–25% B for 40 min, 25%–35% B for 13 min, 35%–90% B for 3 min, and 90% B for 3 min. Peptides were ionized by electrospray ionization at +2.1 kV. Tandem mass spectrometry analysis was carried out on a Q‐Exactive HF‐X mass spectrometer (Thermo Fisher Scientific) under the control of Xcalibur software in a data dependent mode. In each scan cycle, fragmentation spectra of the 25 most intense peptide precursors in the survey scan were acquired in the higher‐energy collisional dissociation (HCD) mode. Full MS scan at Resolution = 60,000, followed by 20 HCD MS/MS scans at Resolution = 15,000, NCE = 27, with an isolation width of 1.6 m/z. The AGC targets for MS1 and MS2 scans were 3 × 10^6^ and 1 × 10^5^, respectively, and the maximum injection time for MS1 and MS2 were 45 and 22 ms, respectively. Precursors of +1, +6, or above, or unassigned charge states were rejected; exclusion of isotopes was disabled; dynamic exclusion was set to 30 s.

### 2.4 Mass spectrometry data analysis

Raw data was processed in the MaxQuant (Version: 2.0.3.0) software environment (Cox & Mann, 2008), and proteins and peptides were identified using the UniProt *C. elegans* reference proteome database (2020). The minimal required peptide length was set to seven amino acids and both protein and peptide identifications were accepted at a false discovery rate of 1%. Database searches were performed according to standard settings with Trypsin/P being used, allowing 2 missed cleavages. Oxidation (M) and Acetyl (Protein N‐term) were allowed as variable modifications with a maximum number of 5. Carbamidomethyl (C) was allowed as a fixed modification. Label‐Free Quantification was enabled, allowing Fast LFQ. The match between runs feature was activated. All other parameters were set as default parameters. Proteomics raw data and selected MaxQuant output files had been deposited to the ProteomeXchange Consortium via the PRIDE partner repository with the dataset identifier PXD039330 (Perez‐Riverol et al., 2022).

### 2.5 Data pre‐processing and one‐way ANOVA analysis

Unless noted, normalization of raw read counts was done using the preprocessCore package (v.4.6.0) in R. Based on the raw read counts matrix of all samples, all worm samples were normalized by the quantile normalization function embedded in preprocessCore, finally, the protein expression values were presented in the log2.

We used one‐way ANOVA to analyze the effects of time factors on the highly variable proteins in worm individuals of different ages. We first created the sample expression matrix and annotation matrix. A new function is defined to return the p‐value of ANOVA for each protein before determining the highly variable protein. A data frame was then created to store the p‐value for each protein. Finally, the protein with p‐value less than 0.01 was selected as the high variation protein for subsequent analysis.

### 2.6 Principal component analysis (PCA)

PCA was performed on normalized LFQ intensity values (log2) of 3252 proteins from 101 individual worms. Principal component analysis of the data matrix using the prcomp function in R. 1266 proteins were generated using the normalized raw data excluding individuals with protein expression deficiencies. The proteins were ranked from largest to smallest among individuals using the coefficient of variation, and the top 1000 proteins were taken as input to apply principal component analysis. We also performed PCA analysis on proteomes of aged worms with different mobility.

### 2.7 GO/KEGG analysis

The protein IDs included in UniProt were converted to the corresponding gene Entrez IDs. GO/KEGG analysis of genes with differentially expressed proteins (DEPs) was performed by the R package ClusterProfiler package (v.4.0.5) (Yu et al., 2012), a tool that analyzes functional profiles of gene and gene clusters. It relied on genome‐wide annotation packages (OrgDb) released by the Bioconductor project, org.Ce.eg.db is an annotation database specific to *C. elegans* (9563 genes are annotated). The annotation of the *C. elegans* genome in GO was based on data provided by Gene Ontology: http://current. geneontology.org/ontology/gobasic.obo (with a date stamp from the source of: 2023‐01‐01.) The clusterProfiler package queries the latest online KEGG database through web API to perform functional analysis. GO terms with p‐value <0.05 were considered significantly enriched for visualization.

### 2.8 Trajectory analysis of worms aging process

The state transitions of the aging process of individual worms were estimated using the Monocle2 (Qiu et al., 2017). Monocle2 is originally an analysis toolkit for single‐cell RNA‐seq, but we used protein expression in a similar format as input for the analysis.

Our protein expression matrix was first constructed as a Seurat object through the CreateSeuratObject function. Then, the annotation file and protein name of the sample extracted from the Seurat object were constructed into the CellDataSet object using newCellDataSet function (expressionFamily parameter was processed by negbinomial.size method). Proteins expressed in fewer than ten samples were filtered out after evaluating the dispersion of the CellDataSet expression matrix using estimateSizeFactors and estimateDispersions. We used Monocle's primary differential analysis function DifferentialGeneTest to identify DEPs with a cut‐off value of q‐value less than 0.01. After the samples were downscaled and sorted using Reversed Graph Embedding (method: ‘DDRTree’), aging trajectory was inferred using the default Monocle parameters. The actual ages of individual worms are labeled with different colors. The heatmap accepted a CellDataSet object containing only differential proteins and generates smooth expression curves along the pseudotime. Then, it clustered these proteins and plots them using the pheatmap package, enabling visualization of protein modules that co‐vary across pseudotimes.

## 3 RESULTS

### 3.1 The proteome of single *C. Elegans* during the aging process

**FIGURE 1:**
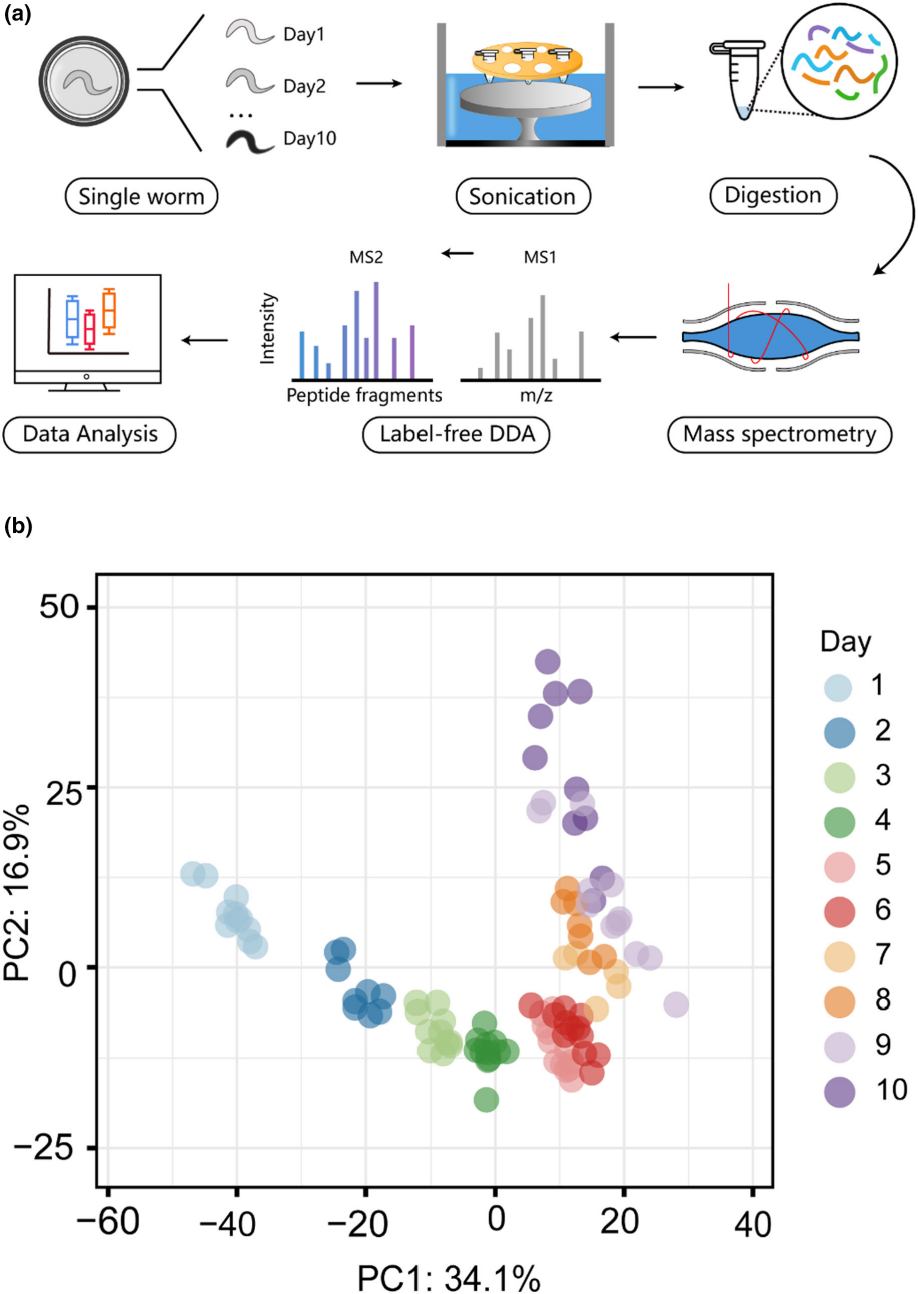
Proteomic analysis of the aging process in *C. elegans*. (a) Schematic of total proteome analysis. Every single worm was manually isolated from adult day 1 to day 10 and then placed individually into a single tube prefilled with lysis buffer. Single worm at different time points were lysed by optimized workflow. After digestion, peptides were analyzed directly followed by nano‐HPLC coupled MS. (b) Principal‐component analysis (PCA) plot of single worms (*n* = 101) from different age groups (represented by different colored dots). The two principal components accounted for 51% of the total. Principal component analysis shows that different individuals of the same age cluster together in the two‐dimensional plot. Different ages have been ordered chronologically in the first principal component of the PCA plot.

Although aging in *C. elegans* is marked by various age‐related phenotypes and gene expression changes, little is known about the variation of molecular features and aging in genetically identical worms. To measure the quantitative proteome of single worms, we optimized a lysis workflow to allow proteome extraction and quantification in single worms (Figure 1a). We quantified on total of 3524 proteins in 157 single worms, ranging from the age of adult day 1, when worms reach adulthood, to adult day 10, when worms have shown apparent aging phenotypes and death events (Table S1, S2). The protein abundances spanned five orders of magnitude and covered major *C. elegans* biological processes and pathways (Figure S1).

The proteomes of single worms at different ages have been ordered chronologically in the first principal component of PCA plot (Figure 1b), suggesting the single‐worm proteome dataset could display both age‐related proteomic changes and inter‐individual proteomic variations, and provide a continuous view of the aging process in *C. elegans*. The first principal component (PC 1) explains age‐related proteomic differences from adult day 1 to day 5, and the second principal component (PC 2) explains proteomic differences from adult day 6 to day 10. PC 1 and PC 2 collectively account for more than 50% of the proteomic variation in our data. The hierarchical clustering results show two large tree branches, one covers samples from adult day 1 to adult day 4, and the other consists of samples from adult day 5 to adult day 10 (Figure S2a), suggesting that the *C. elegans* aging process is not at an even pace across ages.

### 3.2 Pseudotime analysis revealed a reconstructed *C. Elegans* aging trajectory

**FIGURE 2:**
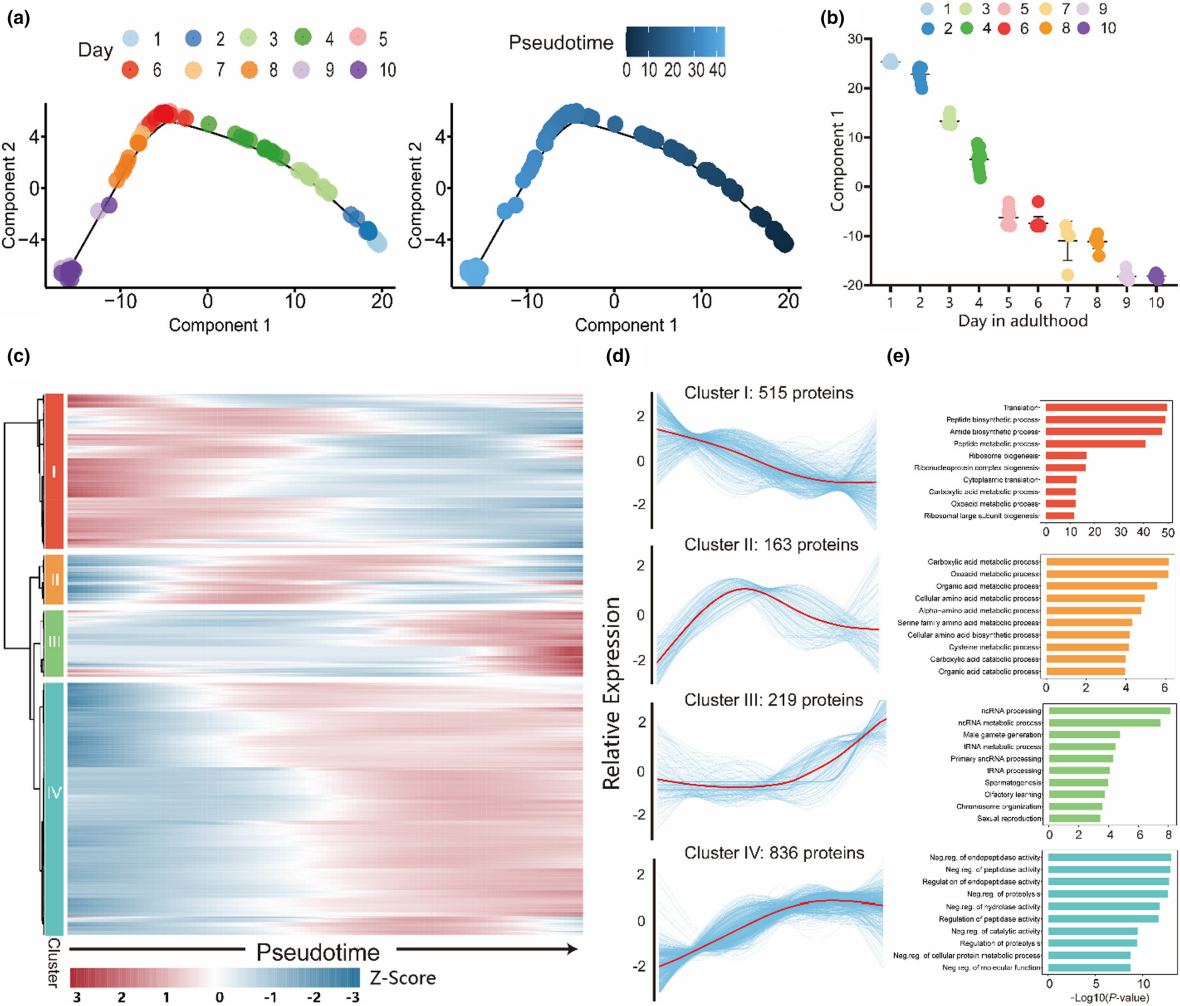
Differential protein expression profiles along worm aging. (a) Pseudotime showing 1000 differentially expressed proteins (DEPs) reconstruction and developmental trajectory of worms (*n* = 101) from different age groups inferred by Monocle 2. Each point corresponds to a single worm. (b) Component 1 of the pseudotime analysis in (a) corresponds to the different ages of individual single worms. (c) Gene expression heatmap of 1733 DEPs (q‐value <0.01) along the pseudotime (columns) was clustered hierarchically into four clusters. The color key from blue to red indicates relative expression levels from low to high. (d) The expression dynamics of 1733 DEPs in (c) were cataloged into 4 major clusters based on trend profiles in a pseudotime manner. Thick lines indicate the average gene expression patterns in each cluster. (e) Gene ontology analyses of each gene cluster in (d). The top ten gene clusters with *p*‐value <0.05. Neg. reg., negative regulation.

To reconstruct the continuous aging process, we have performed pseudotime analysis. Consistent with the PCA result, the proteome of individual worm changes gradually and chronologically across the pseudotime trajectory (Figure 2a). Moreover, there is no apparent branch in the pseudotime backbone, suggesting that although the aging rate varies greatly, the genetically identical individuals generally follow a same aging trajectory. According to the trajectory analysis, the aging process in *C. elegans* could be divided into 3 stages, the first stage (adult day 1–day 5) was marked by dramatic age‐related changes, the second stage (adult day 6–8) was marked less dramatic age‐related changes, while age‐related proteomic changes in individuals in the last stage (adult day 9–day 10) seem to reach a plateau (Figure 2b). The aging stage transition and the deacceleration of age‐related changes were also apparent in the PCA plot and hierarchical clustering results (Figure 1b and Figure S2).

A total of 1733 DEPs across the pseudotime trajectory have been identified at an adjusted *p*‐value <0.01, which shows continuous changes during the aging process (Figure 2c; Table S3). The dynamic changes of these proteins provide insights into the physiological aging state of an individual. The DEPs could be divided into four major clusters according to their expression patterns across the pseudotime, each cluster of proteins corresponds to different aging stages and functional categories (Figure 2d, e). Proteins in cluster 1 were enriched in mRNA translation process and showed decrement during aging, consistent with previous reports. Proteins in cluster 2 show an increment in carboxylate metabolism from the young to mid‐age, and a decrease in elder ages. Proteins in cluster 3 were enriched in RNA metabolic processes and P granule. Proteins in cluster 4 show a gradual increment in proteolysis and the engagement of lysosome in the proteomic changes in aged worms. Collectively, these data suggest that the progression of aging is not linear, instead, the aging processes undergo several stages with distinct age‐related changes.

### 3.3 The proteomes of fast‐aging individuals

**FIGURE 3:**
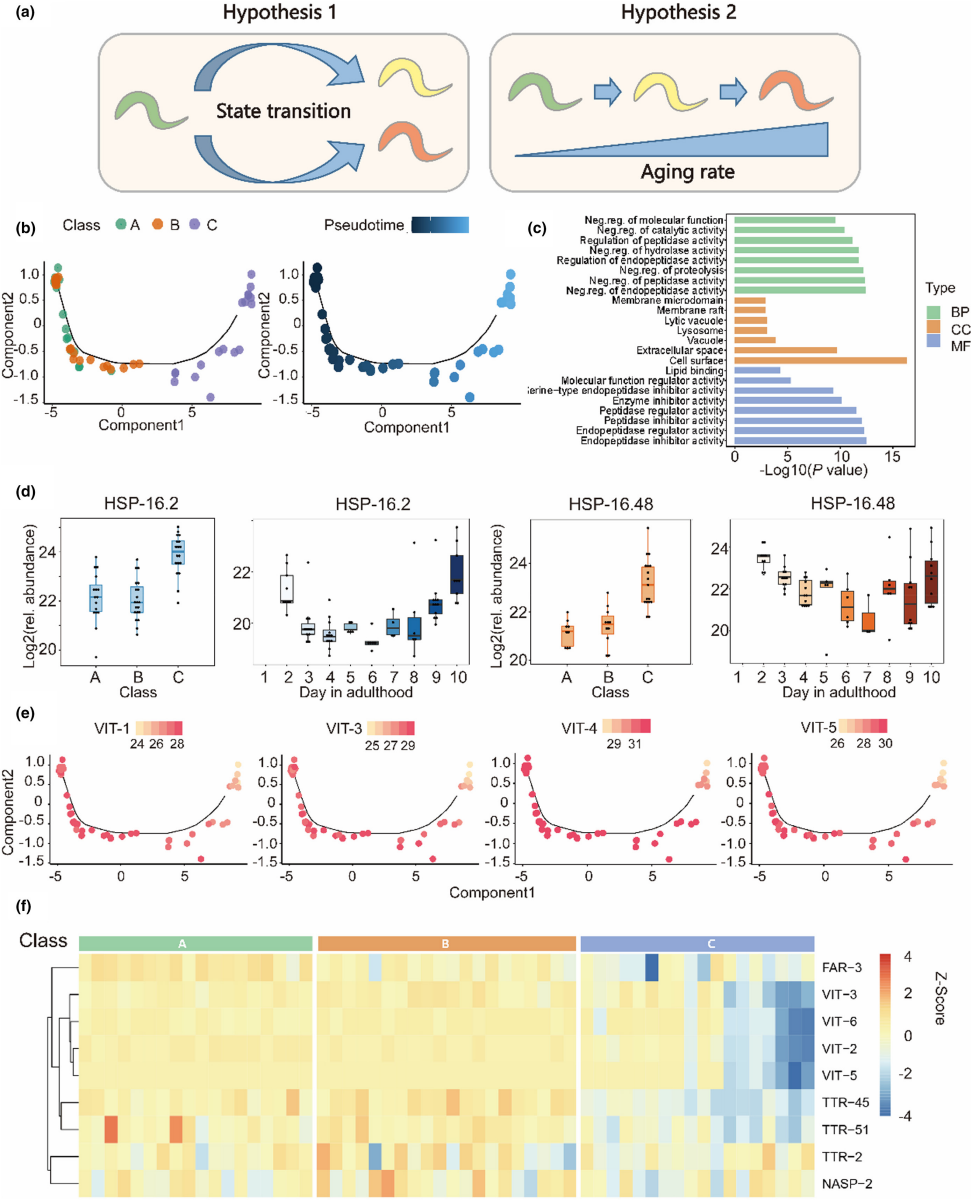
Differential protein expression profiles of worms with different rates of aging. (a) Schematic diagram of two hypotheses of aging trajectory. Young individuals (green); fast‐aging individuals (red); slow‐aging individuals(yellow). (b) Pseudotime showing the 212 DEPs proteins (q‐value <0.01) reconstruction and developmental trajectory of worms (*n* = 56) from day 10 age groups. The Class A, B and C animals, designated based on motility as previously described (Herndon et al., 2002). Each point corresponds to a single worm. (c) Schematic representation of the GO term enrichment analysis of the 212 DEPs proteins (*p*‐value <0.05). BP, biological process; CC, cellular component; MF, molecular function. Neg. reg., negative regulation. Source data are provided in table S4. (d) Boxplots of normalized expression (log2) of significant differentially expressed genes of HSP‐16.2 and HSP‐16.48 respectively, identified in (b) across different age groups. Each black dot represented a single worm. (e) The Dynamic relative abundance (log2) of VIT‐1, VIT‐3, VIT‐4 and VIT‐5 at multiple time points are ordered according to their pseudotime in (b). Each dot represents a single worm. Colors indicate the log2 transformed expression abundance of selected proteins. (f) The hierarchical clustering heatmap showing the Z‐score scaled for the abundance expression of the 9 proteins were published (Kern et al., 2021) including the expression of insulin/IGF‐1 signaling (IIS) and age‐upregulated secreted proteins in Class A, B and C in day 10.

One of the mysteries of aging is about the aging trajectory of fast and slow‐aging individuals. One hypothesis is that the fast and slow‐aging individuals follow different aging paths (Figure 3a), an alternative hypothesis is that the fast and slow aging share the same state transitions but differ in rate. We picked up worms with different movement rates at adult day 10, and classified them into three classes according to previous criteria (Herndon et al., 2002). Briefly, Class A worms are fast‐moving individuals whose remaining lifespan will be quite long, Class B worms may move upon stimulation while leaving a non‐sinusoidal track, and Class C worms are immobile and die within a few days (Figure S3 a, c, d).

By pseudotime analysis, we found that the three classes of worms follow the same clear state transition trajectory (Figure 2a), although the Class C individuals have distinct proteome compared to the other two classes (Figure 3b, Figure S3b,f), the three classes of worms followed the same pseudotime trajectory across the whole aging process, without any branch (Figure S3e), suggesting that different worm individuals share the same state transition during the aging process, despite their difference in the aging rate (Hypothesis 2). Besides, the proteome of Class A and B worms are not distinguishable by either PCA plot or the pseudotime analysis (Figure S3b,f), suggesting that the differences of motility ability between these worms are not characterized by global proteomic changes. We identified 212 proteins differentially expressed across the pseudotime trajectory under an adjusted p‐value <0.01. GO term enrichment analysis revealed that these proteins are enriched in lysosome, cell membrane and extracellular space, suggesting that lysosome mediated degradation of cellular components is a common feature of fast‐aging individuals (Figure 3c; Table S4). We also compared the proteome of Class C versus the Class A/B worms, and identified 42 up‐regulated and 104 down‐regulated proteins (Figure S4a,b; Table S5). The Class C worms are characterized by high levels of lysosomal proteins aspartyl protease ASP‐1, V‐ATPases VHA‐12 and VHA‐20, and other lysosomal membrane protein LMP‐1 (Figure S4c). Although early life enhanced autophagy is beneficial to longevity, autophagy eventually becomes dysfunctional, and harmful to longevity at old age (Wilhelm et al., 2017). Interestingly, we also found the high levels of HSP‐16.2 and HSP‐16.48 in the Class C worms (Figure 3d), as the high expression of HSP‐16.2 in early adulthood predicts a longer lifespan. Here, high levels of these small heat shock proteins may suggest a collapse of proteostasis in the fast‐aging individuals. Thus, the proteomic changes in faster aging individuals suggest that early life beneficial events may play opposite role in elder age to longevity.

*C. elegans* gut to yolk biomass conversion causes multiple senescent pathologies (Ezcurra et al., 2018), and the increment of vitellogenin protein levels during aging has been observed at the mixed population level. Interestingly, we found that the vitellogenin protein levels increased with aging but decreased very rapidly in some individuals when death events start to appear in the population (Figure S5). Additionally, we found that Class C individuals were characterized by lower vitellogenin protein levels (Figure 3e). *C. elegans* vented yolk proteins in the post‐reproductive period, we found that proteins in the vented yolk proteome are lowly preserved in the Class C individuals (Kern et al., 2021) (Figure 3f), suggesting that exhaustion of biomass conversion could be the cause of early death in the fast‐aging individuals. Thus, gut‐to‐yolk biomass conversion followed by yolk venting could be a programmed event that benefits offspring development while promoting aging.

### 3.4 Inter‐individual proteomic variation during aging

**FIGURE 4:**
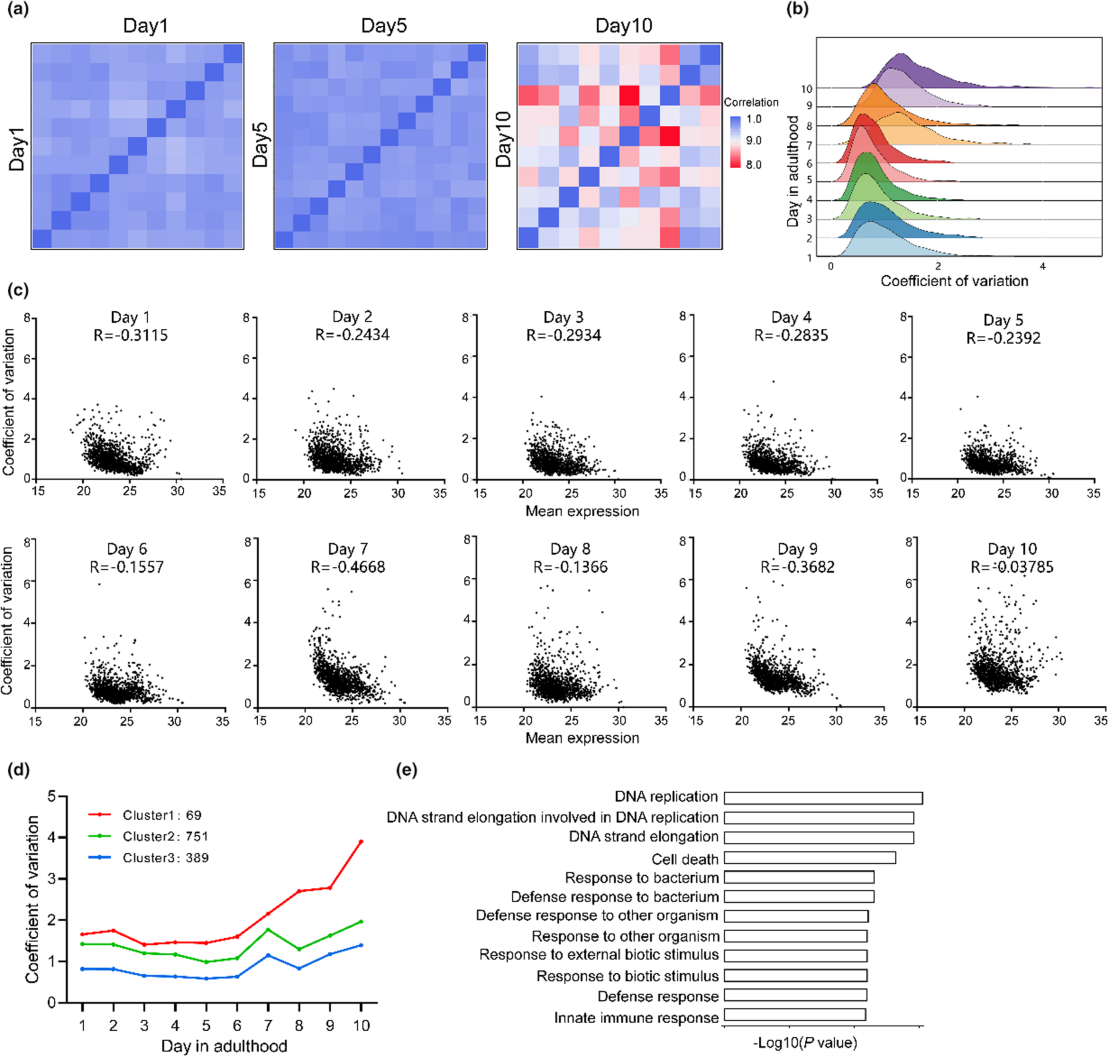
Inter‐individual proteomic variation during aging. (a) Heatmap showing pairwise proteome Spearman correlation values from young (day 1), mid‐age (day 5) and aged (day 10) single worms. (b) Frequencies distribution of protein coefficients of variation (CV) in worms of the same age, with increasing CVs as aged. (c) The pearson correlation between inter‐individual proteomic variation and protein abundance among individuals of each age from day 1 to day 10. The correlation decreases with increasing age. (d) k‐means clustering on the CV of proteins expression abundance in worms of the same age from day 1 to day 10. (e) Enriched GO terms of proteins in cluster 1 in (c). (p‐value <0.05).

To investigate if aging causes increased proteome heterogeneity, we calculated pairwise protein correlation in young, mid‐age and aged worms and found between‐individual proteome correlation decreased during aging, indicating an increased proteome heterogeneity among individual worms (Figure 4a). Coefficient of variances (CVs) of protein abundances among individuals in the same age also increased with age, especially when worms reach the second age stage (From adult day 5) (Figure 4b, c). In adult day 10 individuals, 23.5% of proteins have an inter‐individual CV >2, independent of their abundances (Figure 4c). Highly variable proteins during the aging process were identified by one‐way ANOVA. K‐means clustering on these CV values of highly variable proteins revealed that most of the proteins with an age‐related CV change show increased CVs, while almost none of the protein CV decreases with age. The proteins in cluster 1, which shows the most significant CV increment during aging, were enriched in DNA replication, cell death and stress response processes (Figure 4d, e), suggesting these processes could be a major cause of lifespan variability among isogenic *C. elegans*. Collectively, these results suggest that aging causes the proteome heterogeneity among individuals. Notably, the age‐dependent increment of protein CVs mainly starts from the second stage of aging (from adult day 5), suggesting that this stage is essential to the origin of heterogeneity among individual aging. Proteins exhibit similar functions and usually show co‐expression features. We calculated pairwise protein level correlation and found that the protein levels were co‐regulated in days 1–5 samples, and found a loss of protein co‐expression in days 5–10 samples (Figure S6), suggesting that proteomic changes in the early aging stages show more programmed‐like features, and changes in the mid‐ and late‐ aging stages are more stochastic.

### 3.5 The heterogeneity of aging proteome is not entirely stochastic

**FIGURE 5:**
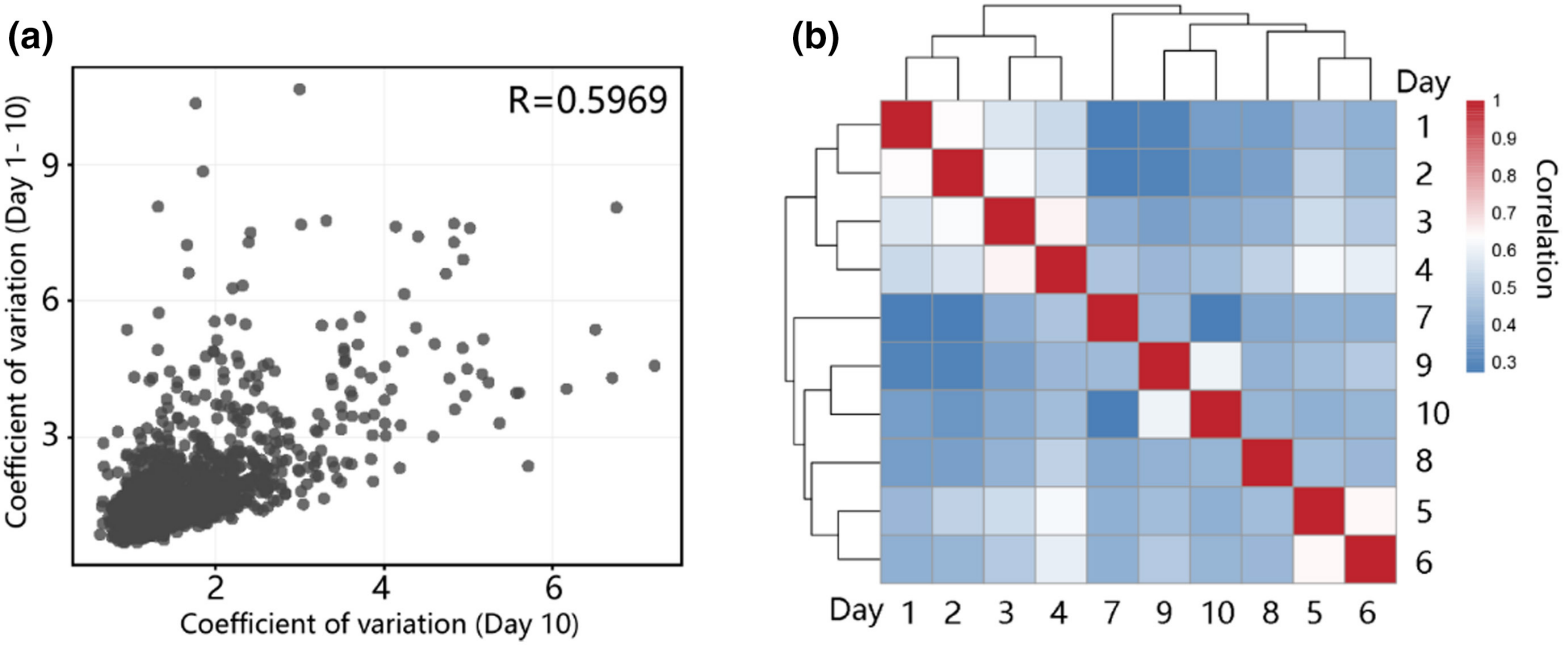
The heterogeneity of aging proteome is not entirely stochastic. (a) The CVs of protein abundance of day 10 individuals correlated with the CVs of protein abundance between individuals across the age range. (b) The heatmap shows the Spearman correlation between the CV of individual worm proteins of the same age and each age group.

The CVs of protein abundance across different ages were compared to CVs of protein abundance across individuals at the same age (Figure 5a; Figure S7). Protein inter‐individual CVs positively correlate with inter‐age CVs (pearson *r* = 0.59), suggesting that the inter‐individual proteomic variations in aged worms are not caused by completely stochastic protein level regulation, instead, a fraction of the proteomic variation originates from age‐related changes that happened unevenly in different individuals. Next, we investigate whether the CV values of proteins of different ages correlate with each other. From day 1 to day 4, the protein CV values show high pairwise correlation and the correlation coefficients are much lower after day 5 (Figure 5b), suggesting that the proteomic heterogeneity is under the control of an unknown ‘program’, and the control of proteomic heterogeneity was attenuated during the aging process.

## 4 DISCUSSION

Despite the tremendous progress made in the genetic regulation of lifespan (Jones et al., 2014; Kenyon, 2010), the inter‐individual lifespan variation among the same species, especially those with the same genetic and environmental conditions, remains a great mystery in aging biology. Genetically identical *C. elegans* cultured in identical environments serve as an ideal model to study the so‐called stochastic heterogeneity of lifespan (Kirkwood et al., 2005). Here, by an optimized single worm proteomic workflow, we analyzed individual proteomic differences of *C. elegans* at different ages and phenotypic states. The *C. elegans* aging process and the lifespan among individuals are highly heterogenous, indicating a substantial of stochastic components that determine aging and lifespan in *C. elegans*. However, the *C. elegans* aging process is not entirely stochastic. The discovery of lifespan regulation genes suggested that the aging process could be controlled by genetic factors (Kenyon, 2010). Besides, a series of molecular and physiological changes during aging seems to happen in a programmed manner (Labbadia & Morimoto, 2015; Li et al., 2019). We believe that dissecting the programmed and stochastic components during aging will shed light on the essential nature of the aging process.

**FIGURE 6:**
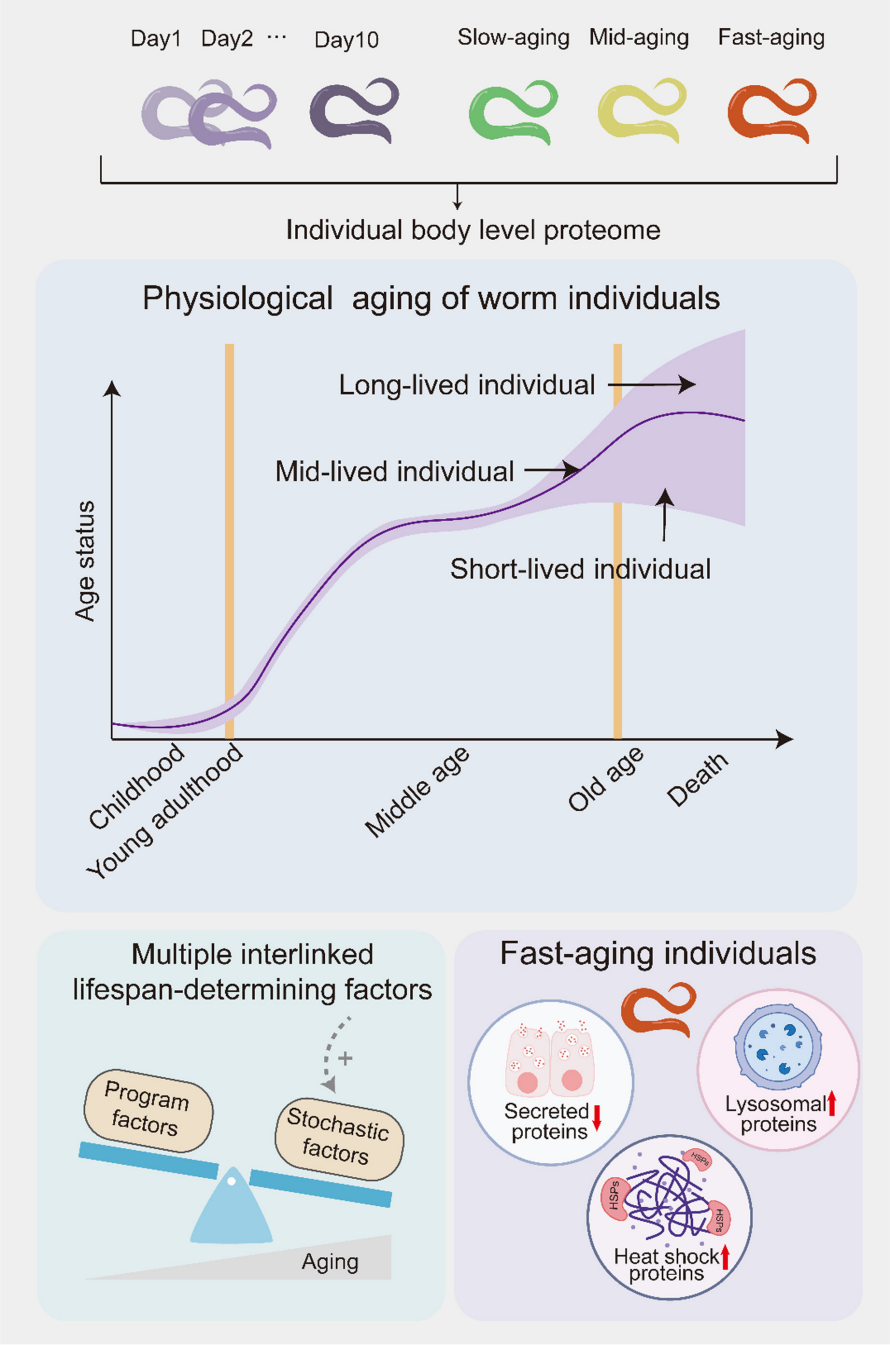
High temporal resolution single worm proteomic landscape reflects the fact that the aging process proceeds nonlinearly. The heterogeneity of the proteome increases during aging. Stochastic factors change increased in later aging stages and programmed factors changes were attenuated. In fast‐aging individuals, secreted proteins were depleted in rapidly, and lysosomal degradation processes and heat shock proteins were overactivated.

Using a high temporal resolution single worm proteomic landscape, we found that the aging process was non‐linearly progressed, with each stage marked by distinct age‐related changes. In addition to factors confirmed previously identified proteins (protein biogenesis, stress response and yolk proteins) that vary in abundance during aging in *C. elegans* (Liang et al., 2014; Narayan et al., 2016; Walther et al., 2015). The single‐worm and high‐temporal resolution of the current dataset enabled us to probe the inter‐individual proteomic variation and detailed dynamics of the age‐related proteomic change. The proteome heterogeneity was increased during the aging process, especially when worms reach mid‐age (adult day 5). Intestine and muscle biomass‐derived yolk milk feeding of larvae by the adult worm is a recently described feature of *C. elegans* life history, the yolk proteins promote offspring development by sacrificing the mother's non‐reproductive tissues (Kern et al., 2021; Turek et al., 2021). Here we found that the fast‐aging individuals are depleted with such secreted proteins and over‐activation of lysosome degradation processes, providing a first molecular link for the programmed enhanced offspring growth and death of the parent individual. This mechanistic link indicates that programmed events in part explain the heterogeneity of adult aging (Figure 6). Our data also provide a molecular‐level perspective on previous studies of the *C. elegans* healthspan. We found that the levels of yolk proteins remain high at mid‐age and dropped rapidly in 1–2 days after adult day 8 in a proportion of individuals, suggesting that the fast‐ and slow‐aging individuals show similar molecular features in early ages and differentiates with each other at about day 9 and 10. The molecular level data are consistent with unproportional healthspan and lifespan changes in recent studies (Bansal et al., 2015; Zhang et al., 2016). Despite the advantages in dissecting the aging process by single‐worm data, the current dataset still suffered from relative low proteomic coverage compared with previous bulk proteomic data, future efforts should be focused on improving the proteomic coverage by optimized sample preparation procedure and more sensitive DIA‐MS approach to quantify more relevant proteins to unravel the heterogeneity proteomes across individuals (Ba et al., 2022).

If we regard the exhaustion of yolk protein production in adult *C. elegans* as a quasi‐programmed event, the relative extension of lifespan in slow‐aging individuals could be resulted from the attenuation of the program by stochastic factors (Figure 6). From this point of view, the evolutionary selection of aging tends to maintain a relatively large proportion of healthspan, and the unproportional extended part of the total lifespan is the result of run‐off the aging program. Consistent with this view, we found that stochastic proteomic variations increased in later aging stages, indicating an attenuation of programmed age‐related changes. The dynamic view of programmed and stochastic factors' contribution to aging also explains the lifespan predictive power of previously reported molecular level biomarkers, biomarkers reflecting ROS levels and hormetic effects are predictive of future lifespan in the developmental stage or early adulthood (Bazopoulou et al., 2019; Knoefler et al., 2012; Rea et al., 2005; Shen et al., 2014), and innate measured stress responsive biomarkers are predictive of future lifespan in isogenic individuals on time‐window in elder ages (Kinser et al., 2021; Sánchez‐Blanco & Kim, 2011).

As discussed above, *C. elegans* are widely used for aging study because of their short life cycle and hermaphrodite characteristics, and genetic tools can be used to identify regulatory pathways for lifespan. Much of the research on how aging is regulated has been found at the genetic level of *C. elegans*. Starting with insulin/IGF‐1 signalling and later the TOR pathway (Kenyon et al., 1993; Vellai et al., 2003), ways to extend lifespan were first described, and then several genes that influence the worm's response to stress and nutrition have been found to affect lifespan. High‐resolution observation and modeling of *C. elegans* lifespan has revealed that multiple causes of aging converged to a single state of ‘biological resilience’ emerges from the interactions of multiple interlinked lifespan‐determining factors (Stroustrup et al., 2016). Although the essence of the biological resilience has not been uncovered until now, reconstructing the aging process into the pseudotime from individual *C. elegans* proteomes at different ages has supported the existence of a sole aging trajectory. Further dissection of such an aging trajectory will provide opportunities for synthetic engineering to utilize the heterogeneity of aging, and to rewire the dynamic change of aging. Of course, there are many difficulties to overcome in transferring relevant research conclusions from simple model organisms to humans. Aging in a specific genetic environment of *C. elegans* may change in another context (Wilson et al., 2020). Human populations have greater genetic heterogeneity. The complexity of biology and the identifiable and unidentifiable variables all influence biological phenotypes. We hope that our study of worm heterogeneity will provide some direction to the future field of precision medicine in terms of individual disease susceptibility and response to drugs.

## AUTHOR CONTRIBUTIONS

Tian‐Yi Zhu and Shang‐Tong Li designed and conducted experiments and data analysis. Dan‐Dan Liu and Lianqi Zhou performed mass spectrometry samples measurement. Xiajun Zhang performed mass spectrometry samples preparation. Rong Zhou and Bing Yang directed the project and wrote the manuscript.

## FUNDING INFORMATION

This work was supported by the outstanding youth fund of Zhejiang Province (LR20B050001), the Chinese National Natural Science Funds (22074132, 91953103 and 31972541), the National Key R&D Program of China (2022YFF0608402), Chinese‐German research project of special program of COVID‐19 (C‐0023), Open Project Program of the State Key Laboratory of Proteomics (SKLPO201806), Open Project Program of Jiangsu Provincial Key Laboratory of Poultry Genetics and Breeding.

## CONFLICT OF INTEREST STATEMENT

All authors claim that there are no conflicts of interest.

## Supporting information


**Table S1.** Quantification information of proteins, differentially expressed proteins and four protein clusters in pseudotime analysis.

**Table S3.** Protein loading scores in PCA analysis.

**Appendix S1.** Supporting Information.

## Data Availability

The mass spectrometry proteomics data has been deposited to the ProteomeXchange Consortium via the PRIDE (Perez‐Riverol et al., 2022) partner repository with the dataset identifier PXD039330.
