# Supplementary material for: Single‐worm quantitative proteomics reveals aging heterogeneity in isogenic Caenorhabditis elegans
Source: Aging Cell. 2023 Dec 3;23(3):e14055. doi: 10.1111/acel.14055 (PMC10928571; doi:10.1111/acel.14055)
Supplement: Supplementary file 3 — Appendix S1. Supporting Information. [file ACEL-23-e14055-s003.docx]

Supporting information


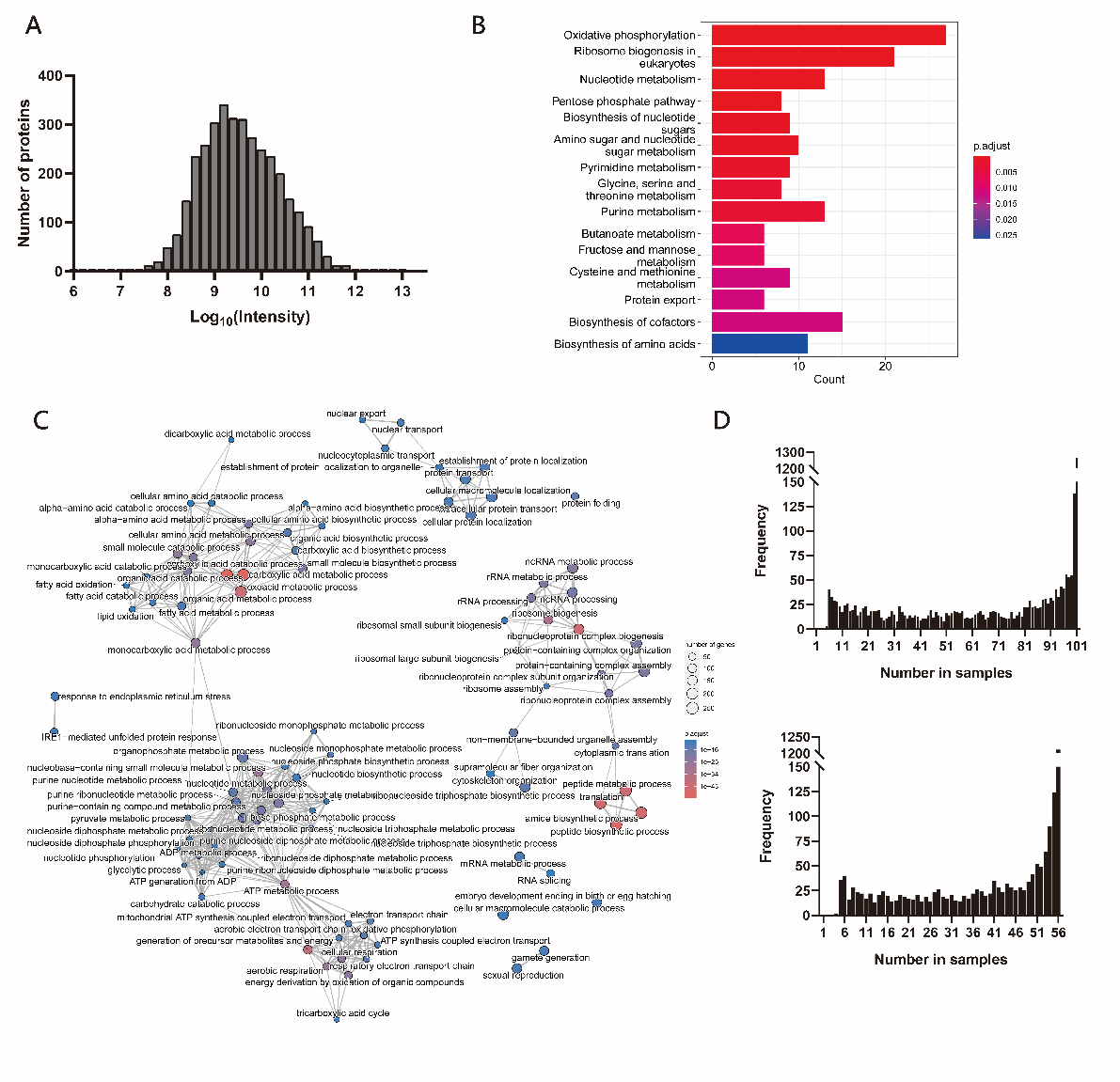


Fig. S1. Assessment of proteomics data quality. (A) Histogram shows the distribution of Log10 transformed protein abundance (MaxQuant LFQ intensity) for days 1-10 the single worm measurements. (B) The bar diagram of the Kyoto Encyclopedia of Genes and Genomes (KEGG) pathway analysis of the total 3,524 proteins identified in all groups. The abscissa represents the enrichment degree. The greater -log10 q-value indicates the higher enrichment degree of the KEGG pathway. The ordinate represents the name of the KEGG pathway. (C) Schematic representation of the GO term enrichment (biological process) analysis of the total 3,524 proteins identified in all samples. The size of the bubbles is indicative of the number of proteins annotated with that GO term (p-value < 0.05). (D) Frequency distribution of identification for each protein. Top: Day 1-10; Bottom: Class A, B and C.


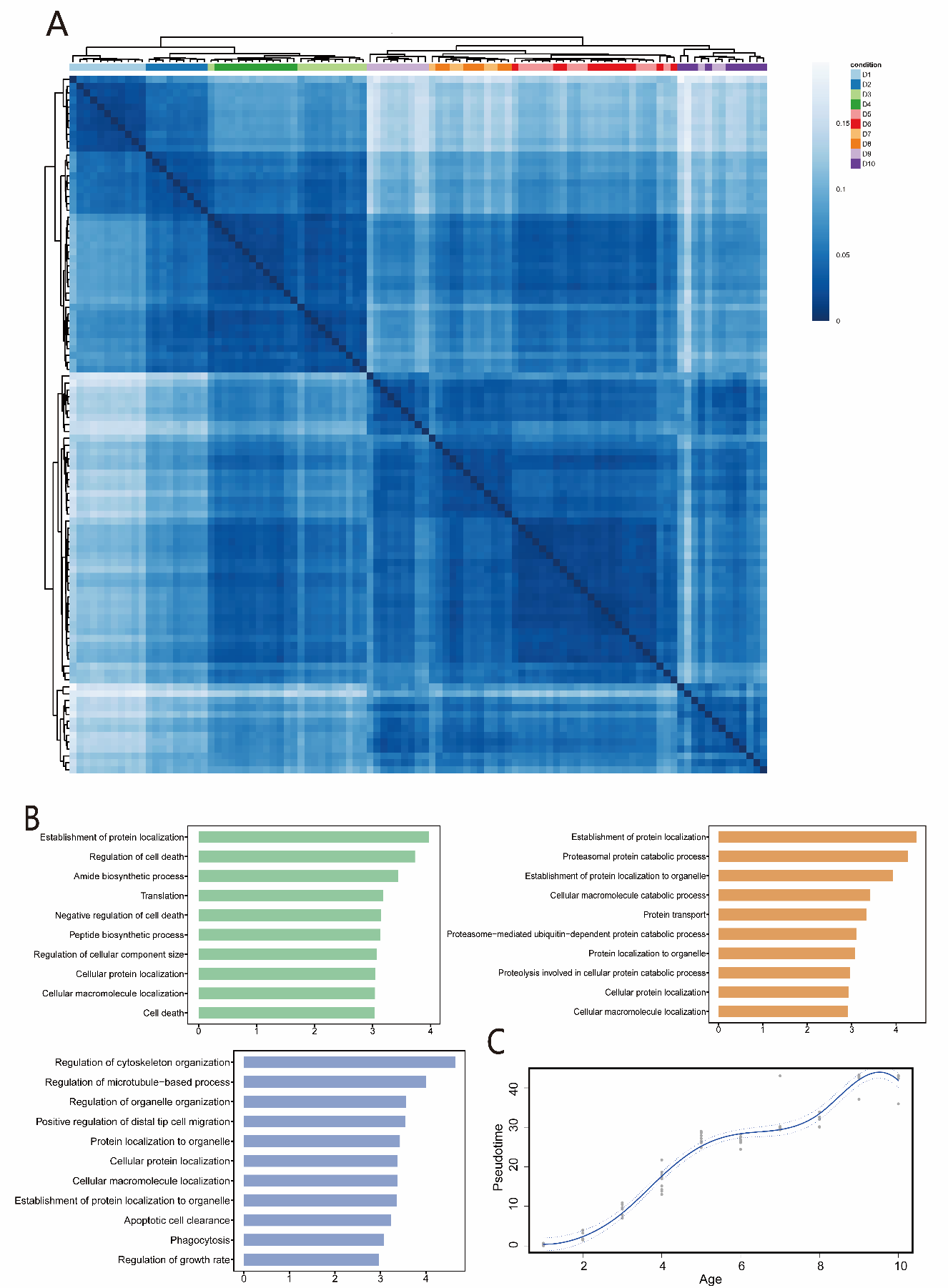


Fig. S2. (A) Heatmap shows the Spearman correlation coefficient between each individual worm. (B) The top 100 proteins with loading scores in each of the three stages of aging classified by PCA analysis were subjected to biological process enrichment analysis of GO. Green: day1-5; Orange: day6-8; Purple: day9-10 (C) The aging curve models of worms of different ages were established by polynomial regression analysis based on the pseudotime results. According to the results of 10-fold cross-validation, polynomial degree=6.


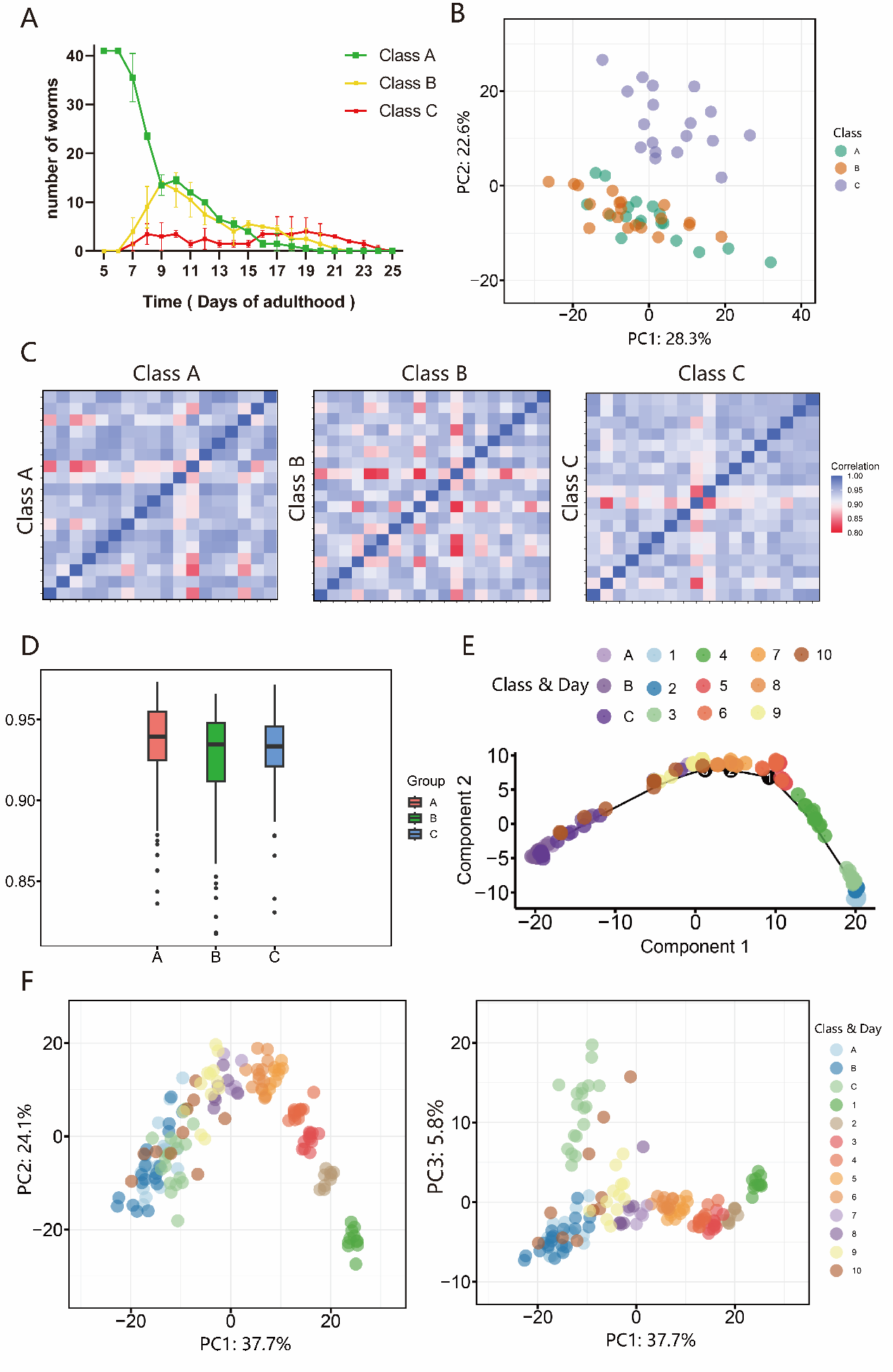


**Fig. S3**. The proteome of Class A and B worms are not distinguishable by either PCA plot or the pseudotime analysis. (A) The lifespan of wild-type N2 worms (n = 41 per experiment) at 20°C was measured; On each day of adulthood, the number of Class A, B and C animals, designated based on motility as described in Herndon et al. 2002, was scored. Shown is the mean ± standard deviation from two independent experiments. (B) PCA plot of single worms (n = 56) from day 10 age group. Different colored dots are used to indicate groups that are distinguished from A, B and C classes by their motility. (C) Heatmap showing pairwise proteome Spearman correlation values from group A, B and C single worms. (D) Boxplot shows the data in the group A, B and C after Spearman pairwise correlation analysis. (E) PCA plot of single worms (n = 157) from all groups. The different colors represent the different age groups and the A, B and C classes from the day 10 age group. (F) Pseudotime showing the 1500 DEPs reconstruction and developmental trajectory of worms (n = 157) from all groups. Each point corresponds to a single worm.


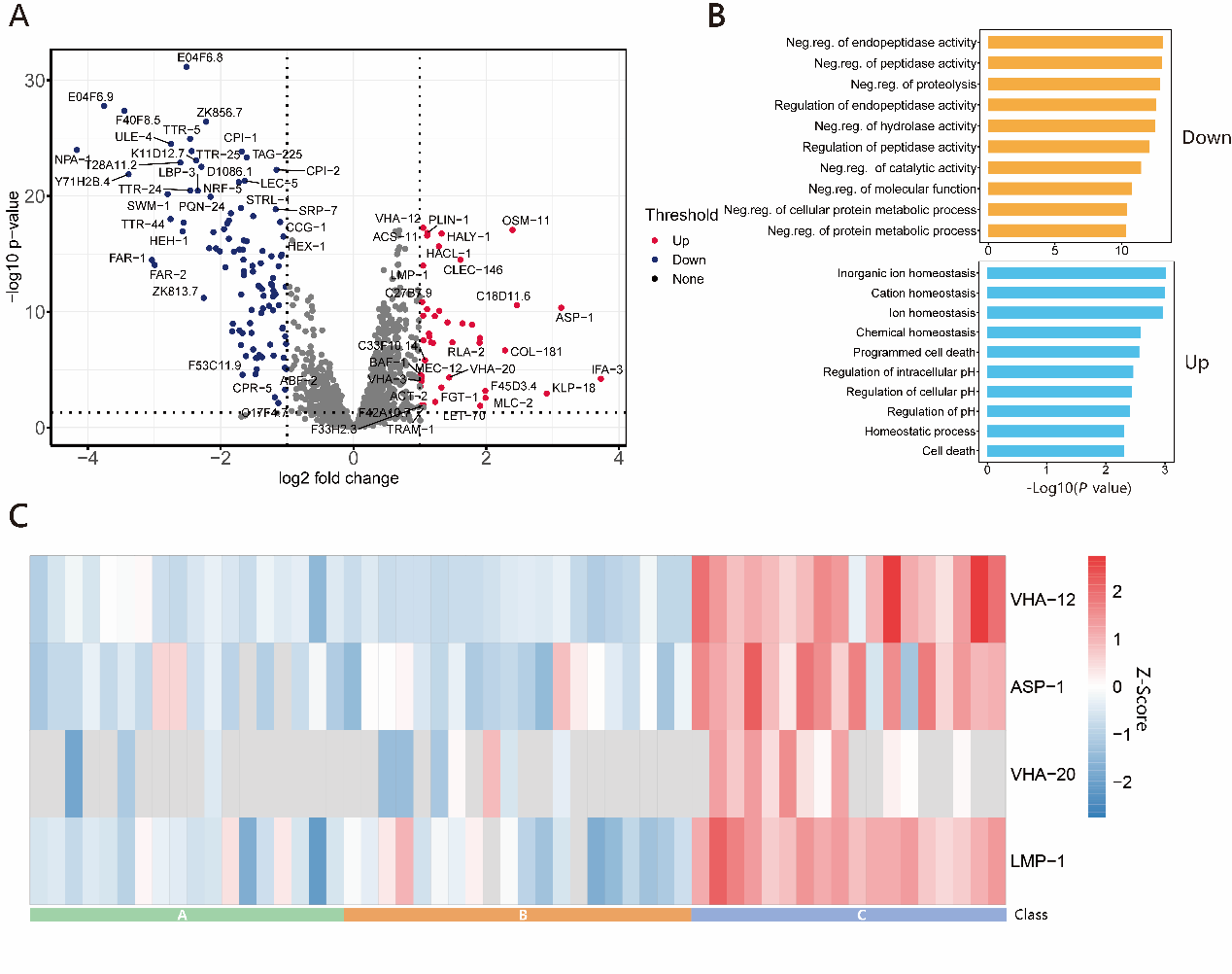


**Fig. S4.** Compared the proteome of Class C versus the Class A/B worms. (A) Volcano plot of relative proteins expression from Class C in the day 10 age group as compared to Class A and B. Red dots significantly upregulated proteins (Fold Change ≥ 2, p-value < 0.05). Blue dots significantly downregulated proteins (Fold Change ≤ 2, p-value < 0.05). grey dots, non-differentially expressed proteins. (B) GO terms for proteins enrichment with up- and down-regulated expression, respectively, in (a). Neg. reg., negative regulation. (C) The heatmap showing the Z-score scaled for the abundance expression of the VHA-12, ASP-1, VHA-20 and LMP-1 in Class A, B and C on day 10. Gray represents missing sample values. (D) Longevity Influence of 146 proteins with up- and down-regulated changes in Class C v.s. AB corresponding to genes that have been reported to affect lifespan.


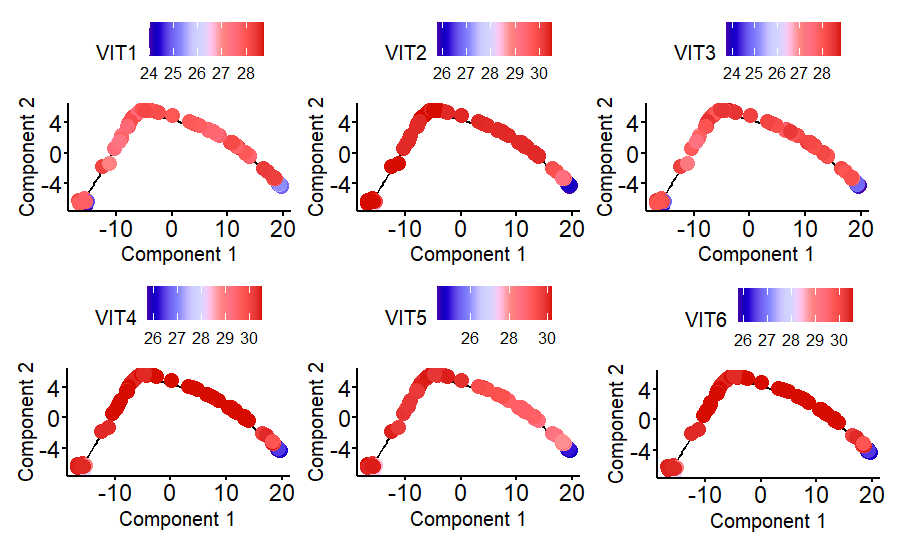


Fig. S5. The dynamic expression of VIT-1-6 at multiple time points are ordered according to their pseudotime from different age groups in Fig.3a. Each dot represents a single worm. Colors indicate the log2 transformed expression abundance of selected proteins.


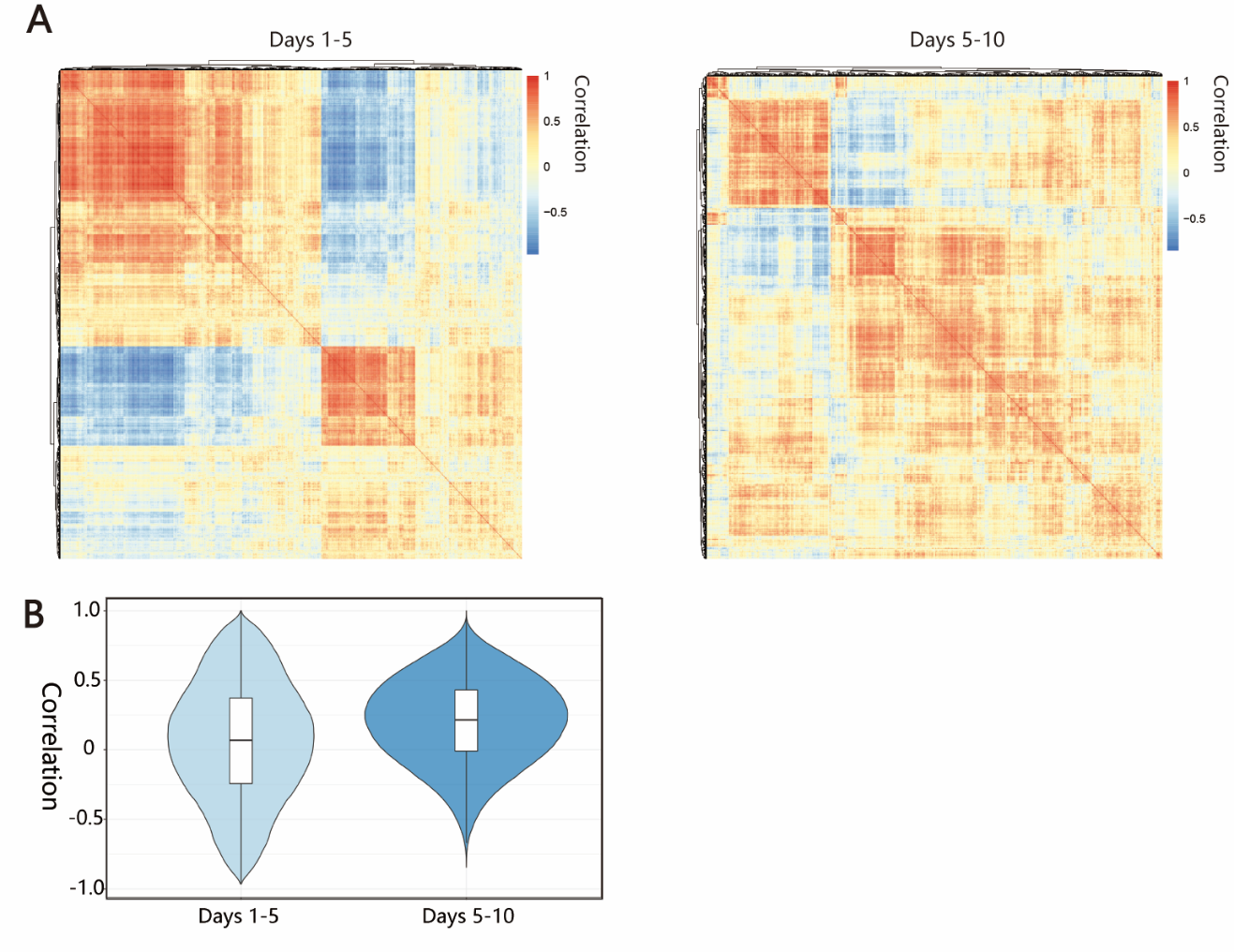


Fig. S6. Paired protein level correlation. (A) The hierarchical clustering heat map showing Pearson correlation coefficients between days 1-5 and days 5-10 worms proteins, respectively. Pearson correlation coefficients were estimated from the abundance expression of 3251 proteins. (B) Violin plot showing the distribution of Pearson correlation coefficients between protein expression of worms on days 1-5 and days 5-10. Pearson correlation coefficients were estimated from the abundance expression of 3251 proteins.


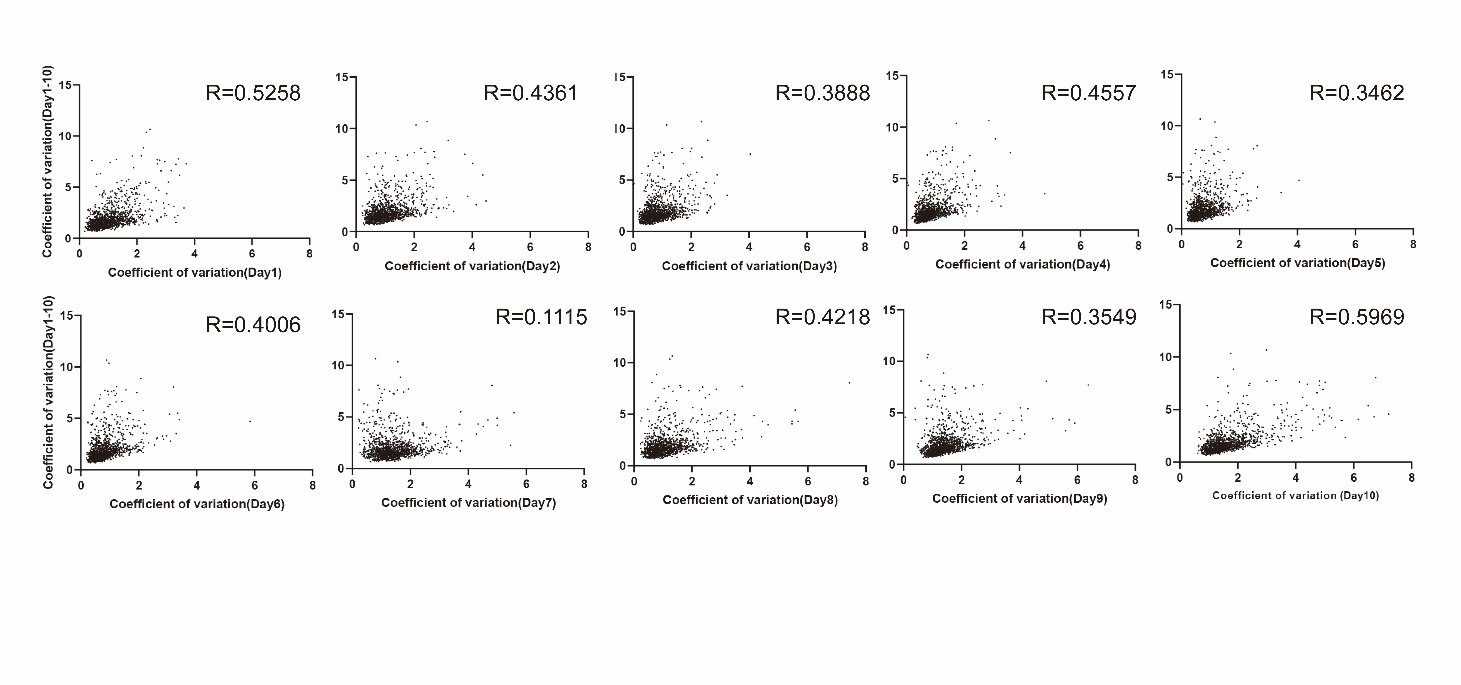


Fig. S7. The CVs of protein abundance at different ages correlated with the CVs of protein abundance among individuals across the age range. Protein inter-individual CVs positively correlate with inter-age CVs.

Table S2. The number of worms in each group in the experiment

| Day in adulthood | 1 | 2 | 3 | 4 | 5 | 6 | 7 | 8 | 9 | 10 |
| --- | --- | --- | --- | --- | --- | --- | --- | --- | --- | --- |
| Number | 11 | 9 | 11 | 12 | 12 | 12 | 5 | 7 | 12 | 10 |

| Group (Day 10) | A | B | C |
| --- | --- | --- | --- |
| Number | 18 | 20 | 18 |

| Description | Ontology | p-value  (-log10) | p-adjust  (-log10) | Fold enrichment |
| --- | --- | --- | --- | --- |
| Negative regulation of endopeptidase activity | BP | 12.37 | 9.75 | 14.28571 |
| Negative regulation of peptidase activity | BP | 12.30 | 9.75 | 14.13043 |
| Negative regulation of proteolysis | BP | 12.16 | 9.75 | 13.81509 |
| Regulation of endopeptidase activity | BP | 11.76 | 9.51 | 12.94821 |
| Negative regulation of hydrolase activity | BP | 11.7 | 9.51 | 12.82051 |
| Regulation of peptidase activity | BP | 11.15 | 9.04 | 11.73285 |
| Negative regulation of catalytic activity | BP | 10.36 | 8.31 | 10.2743 |
| Negative regulation of molecular function | BP | 9.50 | 7.51 | 8.879929 |
| Cell surface | CC | 16.30 | 14.10 | 14.12429 |
| Extracellular space | CC | 9.67 | 7.77 | 6.466513 |
| Vacuole | CC | 3.82 | 2.09 | 4.498715 |
| Lysosome | CC | 3.03 | 1.50 | 4.8583 |
| Lytic vacuole | CC | 2.99 | 1.50 | 4.796163 |
| Membrane raft | CC | 2.86 | 1.50 | 7.751938 |
| Membrane microdomain | CC | 2.86 | 1.50 | 7.751938 |
| Endopeptidase inhibitor activity | MF | 12.47 | 10.29 | 15.20468 |
| Endopeptidase regulator activity | MF | 12.25 | 10.29 | 14.65614 |
| Peptidase inhibitor activity | MF | 12.03 | 10.25 | 14.16122 |
| Peptidase regulator activity | MF | 11.49 | 9.84 | 12.98701 |
| Enzyme inhibitor activity | MF | 10.14 | 8.58 | 10.38339 |
| Serine-type endopeptidase inhibitor activity | MF | 9.34 | 7.86 | 13.31558 |
| Molecular function regulator activity | MF | 5.25 | 3.83 | 3.269978 |
| Lipid binding | MF | 4.28 | 2.93 | 4.639175 |

Table S4. GO term annotation results for 212 DEPs proteins from day 10 age groups

Table S5. Longevity Influence of 146 proteins with up- and down-regulated changes in Class C v.s. AB corresponding to genes that have been reported to affect lifespan

| UniProt ID | Protein | Differential  features | Gene Symbol | Longevity Influence |
| --- | --- | --- | --- | --- |
| Q20615 | Unreviewed | Down | dod-23 | anti |
| H2L2A5 | Unreviewed | Down | F40F8.5 | anti |
| G5EF32 | Unreviewed | Down | npa-1 | anti |
| Q23430 | Insulin-like peptide 7 | Down | ins-7 | anti |
| Q21065 | Intermediate filament protein ifa-3 | Up | ifa-3 | anti |
| Q03575 | Transthyretin-like protein 5 | Down | ttr-5 | anti |
| P55155 | Vitellogenin-1 | Down | vit-1 | anti |
| P06125 | Vitellogenin-5 | Down | vit-5 | anti |
| P05690 | Vitellogenin-2 | Down | vit-2 | anti |
| P02513 | Heat shock protein Hsp-16.48/Hsp-16.49 | Up | hsp-16.48 | pro |
| P02513 | Heat shock protein Hsp-16.48/Hsp-16.49 | Up | hsp-16.49 | pro |
| O44827 | Facilitated glucose transporter protein 1 | Up | fgt-1 | anti |
| Q18947 | Unreviewed | Up | ule-3 | anti |
| Q21049 | Liprin-alpha | Down | syd-2 | pro |
| Q03565 | Barrier-to-autointegration factor 1 | Up | baf-1 | pro |
| O62053 | UPF0375 protein ule-4 | Down | ule-4 | anti |
